# A Rare Cause of Ascites in Preterm Baby: A Case Report

**DOI:** 10.7759/cureus.25577

**Published:** 2022-06-01

**Authors:** Ahmad Mustafa, Farzeen Mohtisham, Adel Sallam, Abdullah Alzahrani, Abrar Ahmed

**Affiliations:** 1 Department of Pediatrics, Neonatology Division, King Abdulaziz Medical City, Ministry of National Guard Health Affairs, Western Region, Jeddah, SAU; 2 Neonatology, King Saud Bin Abdulaziz University for Health Sciences, Jeddah, SAU; 3 Neonatology, King Abdulaziz Medical City Jeddah, Ministry of National Guard Health Affairs, Western Region, Jeddah, SAU; 4 Neonatology, King Abdulaziz Medical City, Ministry of National Guard Health Affairs, Western Region, Jeddah, SAU; 5 Pediatric Intensive Care, King Abdulaziz Medical City, Ministry of National Guard Health Affairs, Western Region, Jeddah, SAU; 6 Department of Neonatology, King Abdulaziz Medical City, Ministry of National Guard Health Affairs, Western Region, Jeddah, SAU

**Keywords:** umbilical catheter, surgery, peritonial drain, imaging, ascites, bladder perforation, urinary catheter, anuria, preterm baby

## Abstract

Bladder rupture leading to urinary ascites in neonates is a very rare occurrence. It can present as a clinical emergency, requiring resuscitation, ventilator support, and acute derangement in renal function. There are only a few reported cases so far in the literature. The commonest etiology is posterior urethral valves which can occasionally lead to urinary ascites even in fetal life. But other proposed etiologies are umbilical arterial catheterization in extreme preterm babies and iatrogenic due to urethral catheterization injuries. Early detection is crucial so that appropriate management, including surgical drainage of the urine if performed early, can lead to the normalization of renal function. Large perforations may need surgical repair after stabilization. These cases can be a challenge for both neonatologists and surgeons. We report a case of bladder perforation in an extreme preterm baby at our hospital.

## Introduction

Bladder rupture leading to urinary ascites in neonates is not a common condition. There are only a few reported cases so far, and the proposed etiologies are posterior urethral valves, umbilical arterial catheterization in extreme preterm babies, and iatrogenic due to urethral catheterization injuries [1]. 

Neonates with urinary ascites usually present as a clinical emergency, requiring resuscitation, respiratory and cardiovascular failure requiring critical care support, and renal impairment [2]. It is a life-threatening condition as the peritoneal membrane "autodialyzes" the urine, leading to a progressive increase in the blood urea nitrogen (BUN) and derangement of the serum electrolytes [3]. Diagnosis is suspected on the basis of ascites with deranged renal function and is confirmed by imaging [4].

## Case presentation

A set of quadruplets were born to a Saudi couple as a result of assisted conception (in vitro fertilization - IVF) at 24 weeks and three days gestation, and the babies were admitted to our neonatal intensive care unit (NICU) for further management.

**Figure 1:**
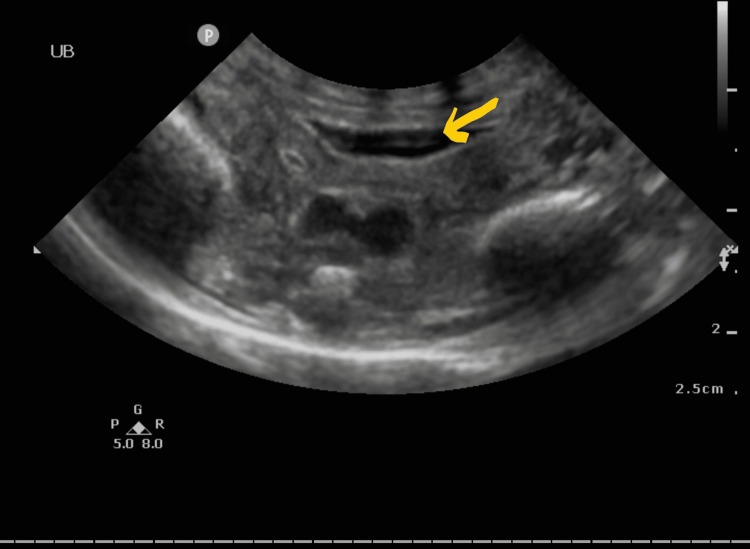
Ultrasound showing almost empty bladder despite the baby being anuric

The second quadruplet weighted 650 grams. He was born by Cesarean section with Apgar scores of five and eight at one and five minutes. He was intubated and ventilated through an endotracheal tube within two minutes of age. Chest X-ray showed moderate respiratory distress syndrome (RDS), and he received two doses of surfactant and was not requiring high ventilatory settings. Umbilical venous and arterial catheters were inserted immediately after birth and verified to be in a good position. He required inotropes from day one for hypotension which were weaned off by the first week of life. He also had metabolic acidosis, which resolved with treatment. The urine output was maintained at around 3-6 ml/kg/hour in the first five days of his life, but after that, the urine output started to decrease, and, eventually, he became totally anuric with rising creatinine and urea, but electrolytes were maintained within normal limits. Attempts to catheterize the bladder failed twice, even with the smallest catheter size of 3.5 French. The urology team was involved, and their attempt at catheterization also failed and led to ecchymosis of the penis with some urethral bleeding. The coagulation profile and platelet count were all normal at that time, and the baby was already off inotropes. The umbilical arterial catheter was still present, and the level was at the ninth thoracic vertebra by X-ray, and the umbilical venous catheter was removed after insertion of the peripherally inserted central catheter (PICC) line. Renal ultrasound done after the failed catheterization attempts showed an empty bladder with mild right-sided hydronephrosis without a hydroureter. There was mild ascites noted at that time (Figure 1). The antenatal ultrasound did not show any significant congenital abnormality in any of the fetuses.

**Figure 2:**
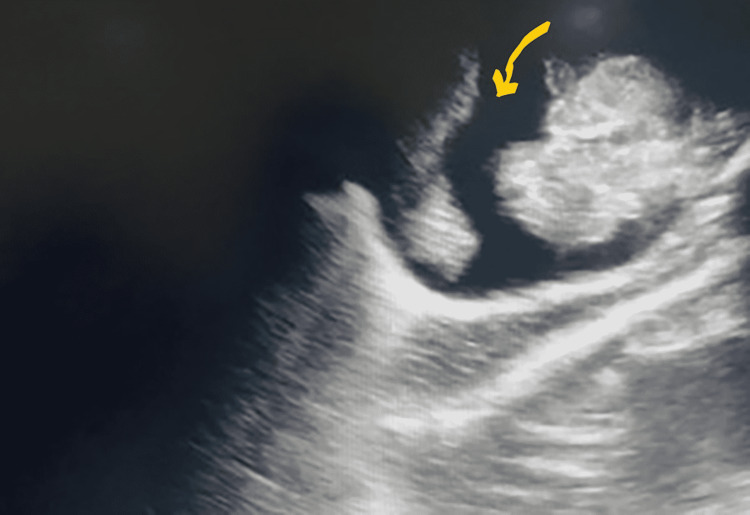
Ultrasound showing severe ascites

Over the next few days, he continued to be anuric with rising urea and creatinine and developed severe ascites, which compromised his ventilation and also caused severe hypotension, which necessitated restarting the inotropes given via the PICC line. There was no pleural effusion or subcutaneous edema. A trial of furosemide treatment both as bolus and infusion failed to drain even a single drop of urine through the urethral opening. Repeated bedside ultrasound again showed an empty bladder with severe ascites (Figure 2).

At that point, urinary ascites was suspected, and a pigtail catheter was inserted to drain the ascites. Immediately after the pigtail insertion, there was a gush of fluid, and almost 100 ml of straw-colored fluid was drained over two hours. The ascitic fluid analysis done on two separate occasions showed urea and creatinine values higher than the serum values (Table 1).

**Table 1:** Laboratory values AGAP - anion gap; BUN - blood urea nitrogen

Parameters	Ascitic fluid	Serum value
Potassium	2.8	3.3
Sodium	138	144
Chloride	100	105
CO_2_	20	19
BUN	33.7	36.5
AGAP	18	20
Ammonia	162	
Creatinine	675	261
Urea	>44.6	>44.6

**Figure 3:**
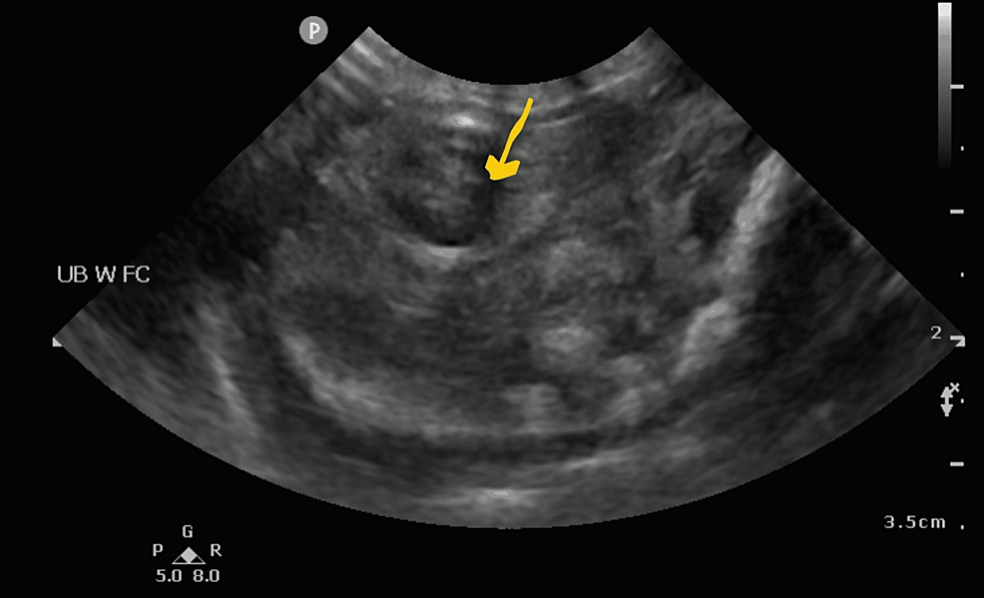
Ultrasound showing resolution of ascites post drainage

His hemodynamic condition improved with continuous drainage of urinary ascites, and he was gradually weaned off from inotropes and shifted back to conventional ventilation from high-frequency oscillatory ventilation. After stabilization, he was operated on at the bedside by the urology team, and a vesicostomy was performed. Clear urine was draining from the bladder, but the bladder perforation could not be clearly delineated. A Foley catheter was inserted into the vesicostomy opening and left in situ. Postoperatively, urine continued to drain from the catheter, the baby's ascites resolved, and the pigtail drain was removed after five days. Also, his renal impairment improved with the return of creatinine back to baseline within a week of surgery. A follow-up abdominal ultrasound showed almost complete resolution of the ascites and significant improvement in the hydronephrosis (Figure 3). He, unfortunately, developed fulminant sepsis nine days later and died within 24 hours despite adequate antibiotics and supportive management.

## Discussion

Rupture bladder in neonates is very rare, but there are case reports mentioning it secondary to renal anomalies, especially posterior urethral valve, which can lead to urinary ascites in fetal life, necessitating fetoscopic surgery for nephrostomy [4]. In extreme preterm babies, spontaneous bladder rupture is reported, and the umbilical arterial catheter is one of the proposed etiologies. Abdominal compression of the bladder to drain the urine and severe urinary tract infection is also mentioned as a cause of rupture bladder [5].

Our baby was extreme preterm with extremely low birth weight. The antenatal ultrasound did not detect any renal anomalies, and he was passing a good amount of urine for the first few days and had a normal renal function initially. But he was hemodynamically unstable since birth and had a stormy course necessitating ionotropic support and hydrocortisone. He had an umbilical arterial catheter, albeit in a good position, had failed bladder catheterization attempts, and also had abdominal compression once to drain the bladder (as he was heavily sedated to comply with high ventilator settings) [6].

So in our case, the cause of bladder perforation was probably multifactorial. Unfortunately, due to extreme prematurity and unstable condition, we could not perform contrast imaging studies on our baby [7]. In fact, even the vesicostomy was done at the bedside due to the same reason.

## Conclusions

Neonatal bladder perforations, even though a rare occurrence, must be considered a differential diagnosis in an anuric baby with isolated ascites. Timely detection and appropriate management are crucial and life-saving in such cases. Ideal imaging to confirm the bladder rupture is a contrast study, but bedside ultrasound can be performed initially. Drainage of the urinary ascites temporarily improves the hemodynamic condition and renal profile, but ultimately surgical management may be needed, especially in case of large perforations. Babies requiring umbilical artery catheterization should be regularly monitored for the catheter tip position, and they should be removed at the earliest when feasible. The urinary catheterization of these extremely preterm babies can be a challenge and must be attempted with the smallest possible catheter and performed by the experts. Abdominal compression to drain urine in such babies is best avoided.
